# Assessing Progress in Reducing the At-Risk Population after 13 Years of the Global Programme to Eliminate Lymphatic Filariasis

**DOI:** 10.1371/journal.pntd.0003333

**Published:** 2014-11-20

**Authors:** Pamela J. Hooper, Brian K. Chu, Alexei Mikhailov, Eric A. Ottesen, Mark Bradley

**Affiliations:** 1 Neglected Tropical Diseases Support Center, Task Force for Global Health, Decatur, Georgia, United States of America; 2 Control of Neglected Tropical Diseases, World Health Organization, Geneva, Switzerland; 3 ENVISION Project, RTI International, Washington D.C., United States of America; 4 Global Health Programs, GlaxoSmithKline, London, United Kingdom; Ministry of Health, Kenya

## Abstract

**Background:**

In 1997, the World Health Assembly adopted Resolution 50.29, committing to the elimination of lymphatic filariasis (LF) as a public health problem, subsequently targeted for 2020. The initial estimates were that 1.2 billion people were at-risk for LF infection globally. Now, 13 years after the Global Programme to Eliminate Lymphatic Filariasis (GPELF) began implementing mass drug administration (MDA) against LF in 2000—during which over 4.4 billion treatments have been distributed in 56 endemic countries—it is most appropriate to estimate the impact that the MDA has had on reducing the population at risk of LF.

**Methodology/Principal Findings:**

To assess GPELF progress in reducing the population at-risk for LF, we developed a model based on defining reductions in risk of infection among cohorts of treated populations following each round of MDA. The model estimates that the number of people currently at risk of infection decreased by 46% to 789 million through 2012.

**Conclusions/Significance:**

Important progress has been made in the global efforts to eliminate LF, but significant scale-up is required over the next 8 years to reach the 2020 elimination goal.

## Author Summary

Lymphatic filariasis (LF) is a widespread neglected tropical disease most frequently recognized as elephantiasis that is caused by parasitic worms and spread by mosquitoes. To overcome this public health problem, the World Health Organization created the Global Programme to Eliminate Lymphatic Filariasis (GPELF) in 2000, subsequently committing to the elimination of LF as a public health problem by the year 2020. Between 2000 and 2012, GPELF provided over 4.4 billion treatments in 56 endemic countries. In order to assess the progress during the first 13 years of GPELF in reducing the at-risk population (initially 1.2 billion) requiring treatment for LF, we developed a model that indicates that the number of people remaining at-risk of infection has decreased by nearly half through 2012 to 789 million people globally. This is important progress in the global efforts to eliminate LF, but significant scale-up is still required over the next 8 years to achieve the 2020 elimination goal.

## Introduction

Lymphatic filariasis (LF) is a Neglected Tropical Disease (NTD) endemic in 73 tropical and sub-tropical countries [1]. It is caused by three species of filarial worms – *Wuchereria bancrofti*, *Brugia malayi* and *Brugia timori* – and is transmitted by multiple species of mosquitoes. The disease manifests as a spectrum of clinical conditions, the most prominent being hydrocele, chronic lymphoedema/elephantiasis of legs and arms, and sub-clinical lymphatic damage, even in young children. Affected individuals suffer from disability, stigma and associated social and economic hardship. Marginalized populations, particularly those living in areas with inadequate sanitation and sub-standard housing conditions are particularly vulnerable and thus most likely to be affected by the disease. According to the initial estimates compiled in 1996, 1.2 billion people were living in areas where they were ‘at-risk’ of acquiring LF and 120 million people were infected, 40 million of whom experienced one or more chronic disease manifestations [2].

In 1997, the World Health Assembly, through resolution 50.29 (WHA 50.29), urged member states and the WHO to capitalize on both new advances in the understanding of lymphatic filariasis and the opportunities for its elimination by developing national plans of action that would lead to the eventual elimination of the disease as a public health problem. In principal, the elimination strategy is relatively straightforward, involving a pre-implementation phase of epidemiological assessment (‘mapping’) followed by a minimum of five annual cycles of once-yearly mass preventive chemotherapy (mass drug administration [MDA]) to all eligible populations residing in geographic zones determined to be endemic. Each cycle of preventive chemotherapy employs a ‘single-dose’ co-administration of two anthelmintics that have impact on adult parasites but are especially effective at reducing microfilariae, the transmission stage of filarial parasites circulating in the blood, to very low levels for periods up to 12 months or more, thereby inhibiting transmission of the microfilaria to mosquitoes. By depleting the reservoir of infectious stages of the parasite across broad geographic areas for five years or more, the transmission cycle of the parasite is expected to be interrupted for long enough to significantly influence parasite population dynamics to the point where elimination is possible [3].

Utilizing a rapid diagnostic test that permits detection of adult filarial worm antigen in daytime blood, national programs first determined the geographic distribution of infection and then embarked on LF elimination [3]. Through early epidemiological assessments and modeling, the initial size of the global population requiring intervention was estimated; the 2013 revised estimate by WHO for the total population living in areas where filariasis had been endemic *when the GPELF began in 2000* (i.e., were ‘at risk’ of infection and, thus, required preventive chemotherapy for lymphatic filariasis) was 1.4 billion [1], [2]. Since the beginning of the program in 2000 through the end of 2012, however, *4.4 billion doses* of anthelmintics had been administered to populations in 56 of the endemic countries, so it is clear that the number of at-risk individuals will have changed [1]. Indeed, the following analysis was conducted to estimate the impact that this global campaign (the Global Programme to Eliminate Lymphatic Filariasis [GPELF]), after its first 13 years, has had on the number of people living at risk of LF infection.

## Methods

### Baseline Data

Baseline at-risk population data in each country were acquired from the publically available Preventive Chemotherapy (PCT) Databank maintained by the NTD unit at WHO in Geneva [4]. The WHO compiles information submitted by endemic countries through their annual program reporting schedule. Early epidemiological assessments were based on determination of microfilariae circulating in the blood, a procedure requiring blood collection at night when the microfilariae are found in the blood but a challenging activity both for field teams and communities alike. The development and adoption of new diagnostics changed the program, such that now national estimates of the population requiring preventive chemotherapy are derived mostly from assessment of parasite-specific antigen prevalence in areas where *Wuchereria bancrofti* is the predominant species, or parasite-specific IgG4 antibody prevalence in areas where *Brugia malayi* or *Brugia timori* predominate. Following earlier experiences from the highly successful LF elimination program in China, mapping in the GPELF was designed to determine whether the population of the ‘Implementation Units’ (IUs – usually health districts) in countries should be considered to be ‘at risk’ of acquiring LF (and, therefore requiring preventive chemotherapy) based on whether there were areas within the IUs where at least 1% of the surveyed population were infected [5], [6]. Different sampling strategies were utilized in different countries, but the total population of the IUs where there were areas of LF prevalence >1% was considered to be ‘at-risk’ and require MDA [6].

### Calculating the Rate of Decline of the At-Risk Population

The recommended WHO strategy to eliminate LF is based on MDA to remove microfilariae from the blood, preventing parasite transmission to mosquitoes. Though programmatic evidence suggests that effective transmission of LF might cease very soon after the initiation of MDA [7]–[9], entomologic studies linked with anti-filarial single dose treatment regimens suggest that the decline in *vector* infection is more gradual [7], [8], [10]–[14]. Our analysis is modeled off this entomologic information coupled with the assumption outlined previously that decreased vector infection leads to a proportionately decreased risk of infection to the endemic population [15], [16]. Thus, the progressive influence of MDA can be estimated by using the progressive decrease in vector infection rates as an indicator for decreased transmission, and, therefore, reduced population at risk of LF. As populations are treated, their risk of infection diminishes progressively after each MDA. Specifically, the available empiric evidence yielded a relationship that describes an ‘average’ rate-of-decline of vector infection as 50%, 25%, 12%, 6% and 0% of pre-treatment levels following each of the first 5 rounds of yearly MDA, a relationship described and utilized in previous studies [15], [16].

### The Model

The population remaining at-risk of infection following a series of MDAs can be modeled *for each LF-endemic country* using the following general algorithm:
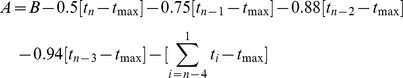
where:

A =  Population still at-risk of infection

B =  Initial population at risk at baseline

T_n_ =  Population treated at n^th^ round of MDA

t_max_ =  Maximum population treated in any round prior to t_n_

n =  Total number of MDAs

**Figure 1:**
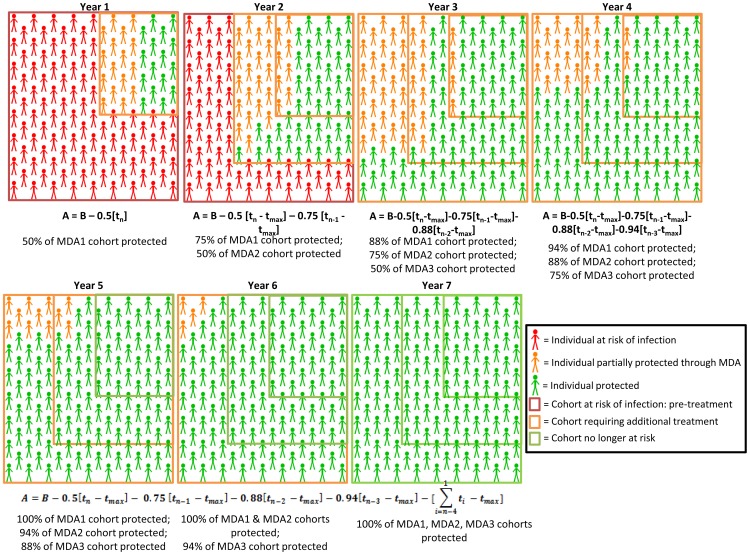
Depiction of progressive scale-up of a national program to full geographic coverage over 3 years. This figure demonstrates the heterogeneous levels of infection risk depending on the number of MDAs experienced by different cohorts of the population defined by when they first received MDA.

Since data at the implementation Unit (IU) level is not available in the PCT Databank for each country, the model builds ‘bottom-up’ from the sub-national to the national level, predicated on the number of *new* persons treated each year (i.e., the treatment cohorts) and followed over time. This approach permits the model to handle staggered MDA rounds on a sub-national level by assigning each cohort its appropriate rate of ‘at-risk’ decline based on the specific number of MDAs it has experienced, rather than applying an *average round* of treatments for the whole country (see Fig. 1).

The model comprises three temporal components:

1. **Before MDA (A = B)**: This part of the model simply states that population still at-risk of infection and requiring treatment is equal to the initial at-risk population at baseline as defined by the WHO PCT Databank (see Assumption 1 below).
2. **During years 1–4 of MDA (B – 0.5[t_n_-t_max_]….0.94[t_n_-t_max_])**: The function [t_n_-t_max_] equals the number of new (i.e., unique) individuals treated in a particular year where t_n_ is the total number treated in the year and t_max_ is the maximum population treated in any prior year (see Assumption 2 below). Therefore, in the initial MDA for any IU, [t_n_-t_max_] equals t_1_ because there is only one MDA and no previous treatment (i.e., t_max_  = 0). Following this first round of MDA, the model presumes that the baseline population at risk of infection is reduced by 50% of the treated population (i.e., 0.5[t_1_]). From MDAs 2–4, the population of this initial treated cohort t_1_ remains the same but the discount factor (‘decreased transmission’) increases to 75% after the second MDA, 88% after the third MDA, and 94% after the fourth MDA.

Meanwhile, the same IU or additional IUs may be scaling up treatment in subsequent years. Again, the model determines only the new population treated in each MDA as [t_n_-t_max_] where t_n_ is the total population treated in the following year and t_max_ is the maximum total population treated in any previous year (t_max_ ≠ 0 after the first year). Each group of [t_n_-t_max_] can now be clearly seen in the model as a mutually exclusive cohort (summed to the national level) based on the number of MDA treatments it has received. This avoids double counting of populations while enforcing proper assignment of infection reduction rates to each treated cohort; the newest ones are closer to the left side of the equation and receive the smallest percent reduction. The model also accounts both for years without new MDA cohorts and for MDAs skipped altogether (see Assumptions 3–5 below).

1. **Year 5 and after of MDA (Σ…)**: The model presumes that any population cohort treated with five or more annual MDAs will see its population *at-risk* reduced by 100% of its pre-treatment level. As a result, the entire sum of these cohorts can be subtracted from the baseline population at risk of infection. The model, however, only reduces the entire at-risk population to zero following programmatic completion of an appropriate evaluation survey (see Figure 1 and Assumption 6 below).

### Key Assumptions

Several key assumptions were made in the formulation of this model:

1. The initial baseline at-risk population for each country is that described in the World Health Organization's PCT Databank [4]. For the 59 countries having at least finished LF mapping, this baseline equates to the maximum annual population in all endemic IUs as defined and reported by the national program for any year *following the completion of mapping*. For the remaining 14 countries that have not yet completed mapping, the Databank's most recent *estimated* population at risk is used as the baseline. This convention leads to an estimated pre-treatment cumulative figure of 1.46 billion people at risk of infection.
2. The model conservatively estimates the number of unique individuals treated over multiple MDAs such that once an individual is treated, it is assumed that s/he is repeatedly treated in subsequent MDAs (so long as the total numbers treated in that country continue to increase). When the annual population treated is higher than that reported from any previous round of MDA (i.e., [*t_n_*-*t*_max_] >0), the difference is classified as *new* individuals treated and added into the transmission decline model as a new cohort.
3. If the annual population treated is less than or equal to that reported from any previous round of MDA (i.e., [*t_n_*-*t*_max_] ≤0), the number of *new* individuals treated for that particular round is considered zero. The model, however, continues to apply the appropriate rate of yearly transmission decline in the existing population cohorts receiving MDA.
4. If a country skips an annual treatment round altogether, the model does not apply any rate of transmission decline for that particular year and instead resumes when the next MDA occurs.
5. The model assumes that treated individuals will not become re-infected while living in MDA-covered areas with overall diminishing LF transmission.
6. Operationally, in any country the population still requiring treatment becomes zero only after a *programmatic decision* to stop MDA as determined by an evaluation survey (most often the transmission assessment survey [TAS]), after which the program is classified by WHO as ‘under post-MDA surveillance’ [17].

## Results

### Estimated Global Achievements (through 2012)

**Figure 2:**
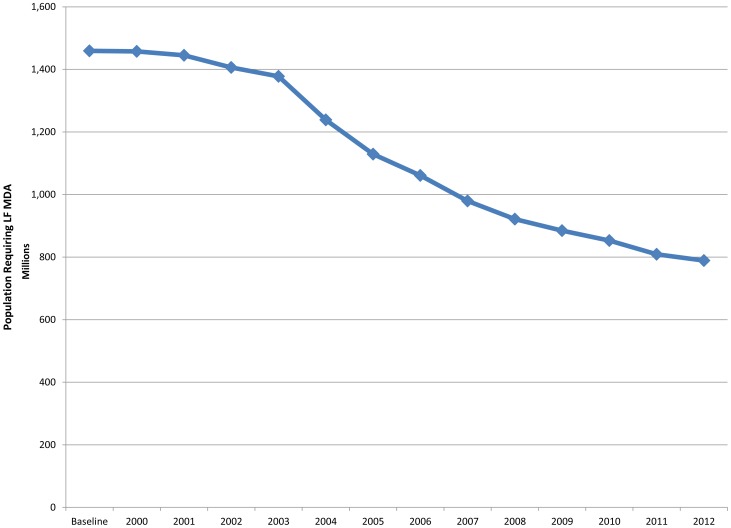
Global decline of population at-risk for lymphatic filariasis. Thirteen years of mass drug administration for lymphatic filariasis have resulted in a 46% decline in the population at risk to 789 million by 2012.

Aggregating the country populations requiring MDA for LF upon completion of mapping (as described above) yields a global total baseline of approximately 1.46 billion people at risk of infection. Applying the model and its assumptions to data from each individual country (schematized in Figure 1) yielded the progressive decline in the at-risk population seen in Figure 2. After 13 years of LF MDA, the global total of 1.46 billion people requiring treatment is estimated to have decreased by 46% to 789 million through 2012.

### Estimated Regional Achievements (Figure 3)

**Figure 3:**
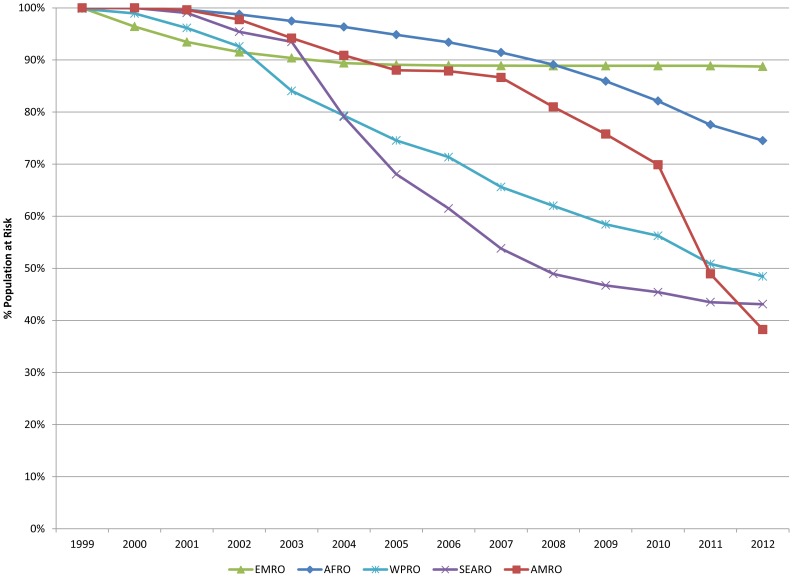
Progressive decline in population at-risk for lymphatic filariasis, by region. Regions have achieved differing levels of progress in reducing the population at risk of lymphatic filariasis.

#### Southeast Asia Region (SEAR)

In SEAR, where the highest burden globally of lymphatic filariasis is found, wide-reaching treatment programs have led to a 57% decrease in the number of people at-risk for LF from an initial at-risk population of over 900 million in 1999 to just over 390 million by the end of 2012. The majority of those still at-risk in this region (over 75% in 2012) are found in India (which has already achieved a decline of nearly 70% through 2012) and Indonesia, which is just now rapidly scaling up its elimination program.

#### Africa Region (AFR)

The African region – with the second highest LF burden globally – still has nearly 350 million people at-risk, a decline of approximately 25% since 2000. The reasons for its relatively slow progress include the inability to treat in *Loa loa* co-endemic areas located in tropical Central Africa, local conflict, and, especially, inadequate financial resources. The highest burden countries in this region – Nigeria, the Democratic Republic of Congo, and Tanzania – represent 50% of the remaining population living in areas at-risk for LF in AFR (as of 2012).

#### Eastern Mediterranean Region (EMR)

Two of the 4 endemic countries in this region – Egypt and Yemen – have completed or almost completed the MDA phase of their LF programs. (Note that as of 2012, South Sudan was still part of the Eastern Mediterranean Region; it moved to the Africa Region in 2013.) Sudan is the highest-burden country in this region (with 92% of the EMR population remaining at risk), and has experienced considerable delays in program roll-out because of recurrent civil unrest.

#### Western Pacific Region (WPR)

Nine WPR countries are currently in a post-MDA surveillance phase of their LF programs, following successful treatment campaigns and reaching the WHO program end-points as determined by a transmission assessment survey. The majority of the remaining burden lies in the Philippines and Papua New Guinea. The Philippines, with 72% of the remaining population at risk in the region, has achieved a 55% decrease in at-risk individuals, but Papua New Guinea currently accounts for 24% of the regional population requiring LF MDA and is yet to achieve scale-up to full geographic coverage.

#### Americas (AMR)

The smallest regional population requiring LF MDA is found in the AMR – just over 4 million people living principally in Haiti, Brazil, and Guyana. By the end of 2012, the region achieved a 62% decline in individuals at-risk of LF infection.

## Discussion

During its first 13 years, WHO's Global Programme to Eliminate Lymphatic Filariasis achieved enormous progress by distributing 4.4 billion treatments in 56 countries and achieving (from our calculations) an estimated 46% reduction in the population at risk of LF from 1.46 billion to 789 million people. By the end of 2012, 13 countries had entered the post-MDA surveillance phase. The available data show that all regions have made substantial progress towards achieving the elimination target of 2020, and for most regions the preponderance of what is left to be achieved is restricted to a few countries with large populations. Overall, these impressive accomplishments were possible only because of strong global partnerships and the commitment of national governments in collaboration with pharmaceutical company partners donating billions of tablets of medicine (Mectizan by Merck & Co., Inc.; albendazole by GlaxoSmithKline; diethylcarbamazine [DEC] by Eisai), bilateral and other donors, non-governmental organizations and researchers, all with guidance and leadership from the World Health Organization. Estimating the number of people freed from the risk of infection is important for all of these key constituencies in order to define the progress being made, while also calling attention to the need to speed the expansion of intervention efforts to ensure that the global elimination targets can be reached.

The accuracy of the estimates from the present model, however, depends on the appropriateness of its underlying assumptions. There is potential both for overestimating the effect of the GPELF in decreasing the number of people at-risk of LF infection and for underestimating it.

A major limitation of this model is its total reliance on the WHO PCT Databank for information on the numbers of people treated each year in the Global Programme. The data is self-reported by national programs, and while in most situations where it has been examined the reported coverage and the independently-surveyed coverage have been similar, there *are* areas where frequent over-reporting has been identified (reviewed in [18]). Such over-reported coverage would lead the model to overestimate the reduction in at-risk population by the GPELF. On the other hand, for the program to be effective, LF MDA guidelines recommend treating at least 65% of the total population in each targeted implementation unit, recognizing that the untreated percentage of the population still garners some level of protection from the treatments in their communities [19]. Since our current model is based on actual treatments distributed, its estimates do not include this additional ‘herd protection’ in populations covered by MDA and, thus could be underestimating overall Programme effects in decreasing the number of at-risk individuals.

Also leading the model potentially to *underestimate* the decrease in numbers of at-risk individuals is the technical convention that the model uses in not ‘zeroing out’ an at-risk population until *all districts in the country* have programmatically passed a Transmission Assessment Survey (TAS) or equivalent. The remaining population at-risk in a country with more than 5 rounds of MDA is likely, therefore, to be overestimated by the model, and the model will show sharp drops in the estimated at-risk population over the next several years as more countries implement the TAS, stop mass treatment, and enter a post-treatment surveillance phase.

The model used in this study also has important additional limitations related to its initial assumptions.

1. **It is a ‘vector-based model.’** The model relies on vector infectivity data post-MDA to estimate LF transmission decrease in human populations using a curve developed earlier and reflected in the integer values of the model's formula [15], [16]. Though data from all three mosquito genera transmitting LF were used to generate the model parameters, the biology of LF infections clearly reflects the characteristics of the vectors, and these differences (whether leading to overestimates or underestimates) are not accounted for currently. Further, extensive advances in vector control interventions in the past decade – particularly in Africa – including the distribution and usage of bed nets, may provide an added benefit of reduced LF transmission; this impact is not currently captured in the model.
2. **Re-infection.** The model does not take into account the possibility of re-infection or resurgence of suppressed infections in areas that miss rounds of treatment, in areas with rounds treating fewer individuals than previous rounds, and in areas achieving consistently poor drug coverage. It is unknown to what extent there could be some level of infection increase in these populations such that they would become an increased risk of infection to others. Since the mosquito infectivity depends on the microfilaremia prevalence in humans, and since the kinetics of the changes in mosquito and human infections post-treatment are not well-defined, it is unclear what interval between successive MDAs in a 5–6 year LF elimination program would lead to increased LF transmission and consequent increased risk of infection to the population.

Despite these modeling constraints, assessing progress of the Global Programme is essential both for demonstrating the successes already achieved and for identifying the challenges remaining. Viewed from the perspective of understanding the population at risk of LF globally, this model has estimated a 46% decrease in those still at risk of acquiring LF infection after 13 years of the GPELF. Clearly, this implies much progress to be celebrated, especially since, for the reasons described above, the ‘true’ reduction in at-risk individuals is likely to be even greater. It is also clear, however, that much more needs to be done – particularly in two important domains. The first is in optimizing MDA drug uptake rates (‘coverage’) in programs already underway, and the second is in extending current programs to those endemic countries (or regions within countries) not already engaged in LF elimination. Where programs have been established, results have been remarkable, but it will be necessary now to maximize program coverage, both programmatically and geographically, in order to meet the global goal of achieving elimination of LF as a public health problem by 2020.
